# The role of attention control in visual mismatch negativity (vMMN) studies

**DOI:** 10.1007/s00221-023-06573-1

**Published:** 2023-03-02

**Authors:** Bela Petro, Zsófia Anna Gaál, Petia Kojouharova, István Czigler

**Affiliations:** 1 Institute of Cognitive Neuroscience and Psychology, Research Centre for Natural Sciences, Budapest, Hungary; 2 Pázmány Péter Catholic University, Budapest, Hungary

**Keywords:** Visual mismatch negativity, vMMN, Behavioral tasks, Attention control, Passive oddball

## Abstract

The detection of unattended visual changes is investigated by the visual mismatch negativity (vMMN) component of event-related potentials (ERPs). The vMMN is measured as the difference between the ERPs to infrequent (deviant) and frequent (standard) stimuli irrelevant to the ongoing task. In the present study, we used human faces expressing different emotions as deviants and standards. In such studies, participants perform various tasks, so their attention is diverted from the vMMN-related stimuli. If such tasks vary in their attentional demand, they might influence the outcome of vMMN studies. In this study, we compared four kinds of frequently used tasks: (1) a tracking task that demanded continuous performance, (2) a detection task where the target stimuli appeared at any time, (3) a detection task where target stimuli appeared only in the inter-stimulus intervals, and (4) a task where target stimuli were members of the stimulus sequence. This fourth task elicited robust vMMN, while in the other three tasks, deviant stimuli elicited moderate posterior negativity (vMMN). We concluded that the ongoing task had a marked influence on vMMN; thus, it is important to consider this effect in vMMN studies.

## Introduction

Information processing of events outside the set of task-related stimuli is important in a complex environment. Electrophysiological methods provide exceptional tools for the investigation of the system capable of dealing with such information. This is because the auditory and visual mismatch components of event-related potentials (ERPs) are signatures of environmental changes without the requirement of overt responses (for a review of auditory mismatch negativity (MMN) and visual mismatch negativity (vMMN) see Fitzgerald and Todd 2020; Stefanics et al. 2014, respectively). MMN and vMMN are usually investigated in the passive oddball paradigm, where within a stream of a frequent stimulus category (standard), there are infrequent (deviant) stimuli from a different category. MMN and vMMN are the ERP differences between the deviant and standard stimuli. To investigate the responses to task-irrelevant events, a usual solution in both auditory and visual modality is the introduction of visual tasks. In the passive oddball paradigm, the task-related events are supposed to prevent attentional processing of MMN- and vMMN-related events.

Recently, auditory and visual MMN is discussed in the predictive coding framework (Friston 2008, 2010; Garrido et al. 2008). According to this framework, the standard events build up a short-term predictive representation of environmental regularities, and in a cascade of processes, the difference between the model and the input as error signal modifies the model. The match between the input and the modified model is the result of perceptual representation of an event. The vMMN is an indicator how this system is working in case of task-irrelevant visual events. Results of vMMN studies using facial stimuli show that this system is capable of dealing with fairly complex events. However, it is important to investigate how much the various methods of diverting attention from vMMN-related events succeed. That is, to what extent do they inform us about the automaticity (i.e., unrelated to attentive processing) of the mechanism proposed by the predictive theory?

Thus, the aim of the present study was to assess the effects of the frequently applied tasks on change detection within the stream of visual events, unrelated to the ongoing task. In vMMN studies (277 items in the WOS database on August 28, 2022), there is a large variety of tasks. In our study, the vMMN-related stimuli were photographs of human faces. We chose these stimuli because (1) it is the most frequently investigated stimulus type in vMMN research (see Czigler and Kojouharova 2022; Kovarski et al. 2017), (2) human faces are highly important stimuli in social interactions.

We investigated vMMN to deviant emotions in human faces. This selection is motivated by the fact, that a search with the term “visual mismatch negativity” in the Web of Science database resulted in 278 items (on September 6, 2022), and 25 percent of the items appeared to be “visual mismatch negativity” AND “face”. Within the face-related studies, we obtained 55 studies on facial emotion.

In the present study, we selected four vMMN paradigms with different sequential and spatial relations to the vMMN-related events. In three cases, the task- and vMMN-related events are spatially separated. In these studies, task-related stimuli are presented to the center of the visual field, and the vMMN-related stimuli appear in eccentric locations. In one set of studies, the task-related stimuli appear in the inter-stimulus interval (ISI) of stimuli of the oddball sequence (for representative studies, see He et al. 2019; Li et al. 2018; Liu et al. 2015; She et al. 2017; Wang et al. 2014; Yin et al. 2018; Zhang et al. 2018). In the present study, this is the ISI task. In other studies, task-related stimuli were presented at any time (i.e., sometimes together with the oddball stimuli; as representative studies, see Amado and Kovács 2016; Fan et al. 2021; Kovács-Bálint et al. 2014; Stefanics et al. 2012). In the present study, this is the ALL task. In the third set of studies, continuous tasks are introduced like visual tracking. For such studies, see Csizmadia et al. 2021; Durant et al. 2017; Heslenfeld 2003; Kojouharova et al. 2019; Sulykos et al. 2018. In the present study, a continuous task was introduced in the TRACK condition. In the ISI task, the participants may discover the temporal separation of task-related and vMMN-related events, and outside the ISI periods, they can observe the stimuli of the oddball sequence. Due to the continuous performance in the TRACK task, participants have less opportunity to observe the oddball stimuli. The FRAME task is a typical three-stimulus oddball paradigm (e.g., Polich and Criado 2006). In the sequence, there are infrequent (standard), infrequent non-target (deviant), and infrequent target stimuli. In such tasks, the stimuli are presented in the center of the screen, and the task requires discrimination between the non-target (standard and deviant) and target stimuli (e.g., Baus et al. 2021; Kask et al. 2021; Kimura et al. 2011; Kovarski et al. 2017, 2021; Sel et al. 2016; Susac et al. 2010). In the FRAME task, the target feature is a colored frame around the facial stimuli. Being members of the same sequence, and there is no spatial separation, participants have the opportunity to observe the vMMN-related stimuli.

It should be noted that, in some study, the task was presented in the auditory modality (e.g., Astikainen and Hietanen 2009; Gayle et al. 2012; Zhao and Li 2006). However, in this respect, the two modalities are not symmetrical. Irrelevant auditory sequences easily become background events, whereas the onset of a sole visual event on the screen attracts attention automatically. In the present study, we do not apply auditory task.

## Method

### Participants

Twenty students (one left handed and one male, mean age = 20.7 years, SD = 2.13) with normal, or corrected-to-normal vision (at least 5/5 in a version of the Snellen charts), participated in the experiment for course credits, who had no known neurological or psychiatric disorder. The reason to choose this sample size was that through the search of the Web of Science database (“visual mismatch negativity”) AND (face or facial) AND (emotion OR emotional OR affective OR affect), we identified 55 datasets (06.09.2022). The average sample size of these datasets was 19.5 (SD = 8.16). Furthermore, according to a G*Power calculation (Faul et al. 2009), it was reported (Chen et al. 2020) that at effect size of *d* = 0.7, at least 19 participants would be required for 80% power to detect the effect with an alpha level of 0.05.

Written informed consent was obtained from all participants prior to the experimental procedure. The study was conducted in accordance with the Declaration of Helsinki and approved by the United Ethical Review Committee for Research in Psychology (EPKEB).

### Stimuli and procedure

The participants were seated in a dark, electrically shielded and sound-attenuated room. The stimuli were presented on a 24-in. LCD monitor (Asus VS229na) with a 60 Hz refresh rate. Participants were seated with their head 1.4 m from the computer screen.

The vMMN-related stimuli were photos of happy and sad faces from the Karolinska Directed Emotional Faces (KDEF) database (Lundqvist et al. 1998). The set of photographs consisted of greyscale, portrait photos of three male and three female models with both expressions. In all four paradigms, the size of the photographs was 7.4 degrees of vertical visual arc and 5.5 degrees of horizontal visual arc, and they were displayed on a light grey background (47 cd/m^2^). The photos appeared quasi-randomly, so that all of them were presented in equal number, and the same face never appeared consecutively. There were four conditions. In the three-stimulus oddball condition (FRAME), the photos appeared in the middle of the screen. In the other three conditions (TRACK, ISI, ALL), two identical photographs were presented on the left and right side of the display with a distance of 5.57 degrees of visual arc between them. The stimulus duration was 150 ms, the inter-stimulus interval was 450 ± 33 ms in all conditions. In the FRAME condition, the participants had to press the space button on a computer keyboard as fast as possible, whenever the photo appeared with a red (0.6, 0, 0 in RGB values, 14 cd/m^2^) frame (30 pixel wide). There were 800 standard stimuli, 100 standards with a frame (target), and 100 deviant stimuli. Framed standards and deviants were interspersed randomly in the standard sequence with at least two standards between targets or deviants. In the other three conditions, 100 deviants were interspersed in the sequence of 400 standards randomly in a similar manner. In the ISI and ALL conditions, the participants’ task was to press the space button of the keyboard as fast as possible, whenever the dimension of the fixation cross (white, 230 cd/m^2^) changed (the length of the horizontal line was 0.66 grade of visual arc and that of the vertical line was 0.33, and vice versa after a change). These changes occurred randomly between 5 and 15 s. In the ALL condition, this change could occur at any time. In the ISI condition, the fixation cross changed only in the inter-stimulus interval. In the TRACK condition, the participants performed a similar tracking task as in the study of Sulykos et al. (2018). In the middle of the screen between the photos, there was a red fixation point (diameter: 3 min of visual arc), with a green disc (‘ball’; diameter: 6 min of visual arc) moving horizontally across this, and the task being to keep this moving ball on the red fixation point using the left and right arrow keys on the keyboard. Maintaining the disc on the fixation point, or no more than 0.4 min of visual arc beyond this was taken to be on the fixation point, as at a further distance, this color changed to blue to indicate an error. This tracking task demanded continuous visual attention on the center of the screen as the direction of the moving disc changed randomly.

Reverse control procedure was applied in all four conditions. That is, the standard and deviant stimuli (happy or sad faces) were interchanged in separate sequences in a counterbalanced order between participants. We split the sequences within each condition into 2.5 min-long blocks to avoid fatigue. These blocks were presented consecutively. So, the whole experiment was 20 × 2.5 min = 50 min long (net recording time). The order of conditions was varied and counterbalanced between participants. The participants received feedback after each block on their performance (mean reaction time, or the number of errors in the tracking task). At the beginning of a new task type, the participants received some verbal information, and instructions were displayed on the screen.

### Measurement and analysis of brain electric activity

Electrical brain activity was recorded from 64 locations according to the international 10–20 system (BrainVision Recorder 1.21.0303, ActiChamp amplifier, Ag/AgCl active electrodes, EasyCap (Brain Products GmbH), sampling rate: 500 Hz, DC-70 Hz online filtering). The reference electrode was on the nose tip, and the ground electrode was placed on the forehead (FPz). Both horizontal and vertical electrooculogram (HEOG and VEOG) were recorded with bipolar configurations between two electrodes (placed lateral to the outer canthi of the two eyes and above and below the left eye, respectively). The EEG signal was bandpass filtered offline with a non-causal Kaiser-windowed Finite Impulse Response filter (low pass filter parameters: 30 Hz of cutoff frequency, beta of 12.265, a transition bandwidth of 10 Hz; high pass filter parameters: 0.1 Hz of cutoff frequency). Stimulus onset was measured by a photodiode, providing exact zero value for averaging. Epochs ranging from − 100 to 450 ms relative to the onset of stimuli were extracted for further analysis. The first 100 ms of each epoch served as the baseline. Epochs with larger than 100 μV voltage change at any electrode were considered artefacts and rejected from further processing. The EEG data were processed with MATLAB R2014a (MathWorks, Natick, MA). ERPs to standard stimuli that preceded deviants were involved to the averaging.

VMMN is expected in the 150–300 ms post-stimulus range as the difference between the ERP amplitudes between the deviant and standard. Deviant minus standard difference potentials frequently consists of an earlier and a later part (e.g., Kimura et al. 2009; Maekawa et al. 2005; Sulykos et al. 2018). Therefore, we divided this epoch into two parts (150–225 and 226–300 ms), and measured the average amplitudes elicited by the deviant and standard at the PO7 and PO8 locations (i.e., the most frequent locations of vMMN to facial emotions). The two epochs were analyzed separately. To analyze deviance effects, we applied the following calculations. The TRACK, ISI, and ALL conditions were different only in the tasks. Therefore, these conditions were compared in three-way ANOVAs with factors of condition (TRACK, ALL, ISI), stimulus (deviant, standard), and side (left, right). Separate two-way ANOVAs with factors of stimulus (deviant, standard) and side (left, right) was calculated for the FRAME condition, because the stimulation was different (photographs appeared centrally) in this condition. Difference potentials of the four conditions were compared in two-way ANOVA with factors of Condition (TRACK, ISI, ALL, FRAME) and Side (left, right). When appropriate, the Greenhouse–Geisser procedure was applied. Effect size was calculated as partial eta square (*ƞ*_p_^2^). In post hoc analyses, Bonferroni test was calculated, and in reported differences, the alpha levels were 0.05. We used the Statistica package (Version 13.4.0.14, TIBCO Software Inc.) for statistical analyses.

We conducted Bayesian analyses (van den Bergh et al. 2020) with the same factors, as suggested by van den Bergh et al. (2020). As an indicator of the evidence for the alternative and null hypothesis, we applied BF_incl_. In these analyses, the JASP (https://jasp-stats.org/) programs were used.

## Results

### Behavioral results

Performance in the tracking task of the TRACK condition was expressed as the number of color changes of the disc, i.e., the incidents when the disc was outside of the target field. The mean of such erroneous events in the four blocks was 0.66 (S.E.M. = 0.14). In the ISI and ALL conditions, the mean reaction time for the occasional changes in the fixation cross was 473 ms (S.E.M. = 12.62) and 471 ms (S.E.M. = 11.98), respectively. In both conditions, there were on average 14.6 cross-flips per block. Participants missed the cross-flips 0.8 times/block (S.E.M. = 0.29) in the ISI condition, and 0.32 times/block (S.E.M. = 0.1) in the ALL condition. This difference between the two conditions is significant, *t*(19) = 2.1, *p* < 0.05, Cohen’s *d* = 0.47. In the FRAME condition, the average reaction time for the appearance of framed picture was 449 ms (S.E.M. = 8.58). There were 24 framed pictures per block, and participants missed 0.84 times/block (S.E.M. = 0.18). Accordingly, performance in the four conditions was fairly high, showing that participants attended to the task-related stimuli. Due to the high performance in the ISI condition, the slightly lower performance in this condition than in the ALL condition should be treated carefully, even if this could indicate that, in the ISI conditions, the saliency of the faces was higher.

### ERPs

**Fig.1:**
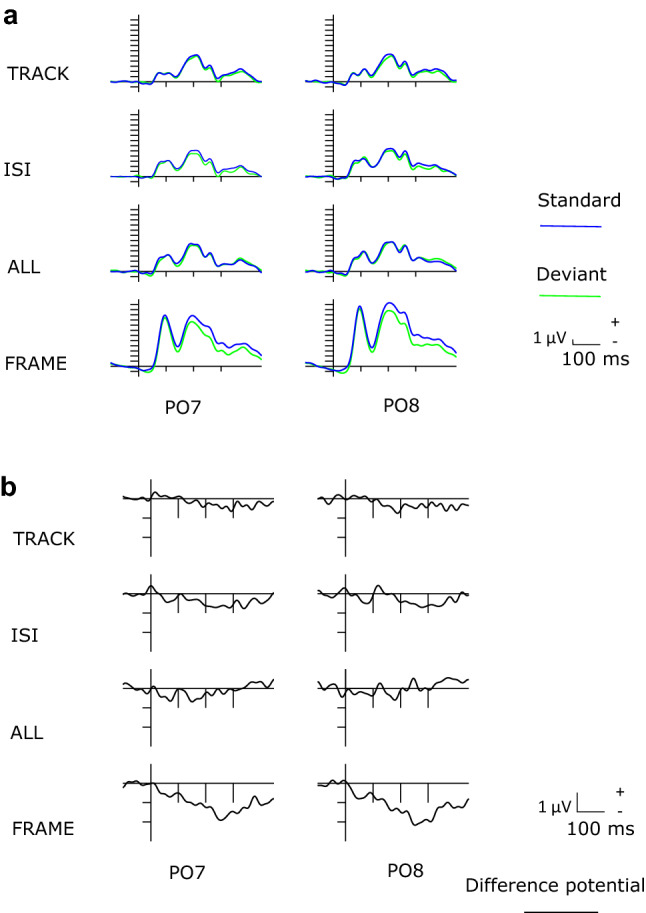
**a** Event-related potentials in the four conditions (TRACK, ISI, ALL, FRAME). **b** Deviant minus standard difference potentials in the four conditions (PO7 and PO8 locations)

**Fig. 2:**
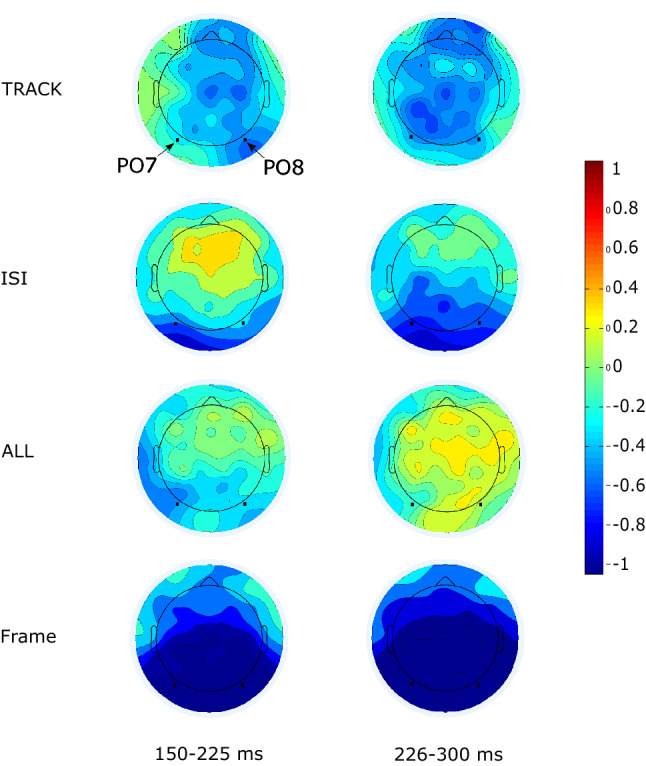
Surface distributions of difference potentials in the four conditions (150–225 and 226–300 ms epochs), PO7 and PO8 electrode locations are indicated with dots

Figure 1 shows the ERPs to the deviant and standard stimuli at PO7 and PO8 locations (a), and the difference potentials at these locations (b). Table 1 shows the mean amplitude of the difference potentials, and Fig. 2 shows the scalp distribution of the difference potentials in the four conditions in the 150–225 and 226–300 ms ranges. As this figure shows, robust negativity emerged in the FRAME condition in both epochs, but deviant stimuli elicited less positivity (i.e., the difference potential was negative) also in all other conditions, with a more typical posterior distribution in the ISI condition. In the TRACK condition, anterior locations also indicated negative difference. However, as Fig. 2 and Table 1 show, the amplitude differences in TRACK, ISI, and ALL conditions were low, it was in the − 0.23 to − 0.49 µV range in the earlier epoch, and + 0.11 to − 0.67 µV range in the later epoch. The ANOVA results corresponded to this notion.

**Table 1:** Mean amplitudes (µV) of the deviant minus standard difference potentials in the 150–225 and 226–300 ms ranges in the four conditions with S.E.M. in the brackets

	Range			
	150–225 ms		226–300 ms	
	PO7	PO8	PO7	PO8
condition				
TRACK	− 0.25 (0.13)	− 0.48 (0.15)	− 0.44 (0.16)	− 0.58 (0.18)
ISI	− 0.49 (0.14)	− 0.23 (0.13)	− 0.67 (0.16)	− 0.60 (0.16)
ALL	− 0.43 (0.10)	− 0.29 (0.11)	− 0.14 (0.11)	+ 0.11 (0.13)
FRAME	− 1.14 (0.13)	− 1.86 (0.15)	− 1.69 (0.13)	− 1.95 (0.14)

In the 150–225 ms epoch, the three-way ANOVA on the TRACK, ISI, and ALL conditions resulted only significant main effect of stimulus, *F*(1,19) = 4.88, *ƞ*_p_^2^ = 0.20, *p* < 0.05 (3.94 vs. 4.30 µV). However, the Bayesian analysis provided only anecdotal evidence of this difference, BF_incl._ = 1.790. Concerning the differences among the conditions, there was moderate evidence for the null hypothesis, BF_incl._ = 0.195. In the twoway analysis on the FRAME condition, the main effects of stimulus and side were significant, *F*(1,19) = 25.34, *p* < 0001, *ƞ*_p_^2^ = 0.57 and *F*(1,19) = 5.24, *p* < 0.05, *ƞ*_p_^2^ = 0.22. The Bayesian analysis resulted in similar results, it provided moderate evidence for the stimulus difference, BF_incl._ = 5.629, and very strong evidence for the lateral (side) difference, BF_incl._ = 63.579, i.e., deviants elicited less positive ERPs (7.94 vs. 9.21 µV, and the ERPs were larger on the right side (9.44 vs. 7.71 µV).

The two-way ANOVA on the difference potentials of the four conditions resulted significant condition main effect, *F*(3,57) = 3.38, *ɛ* = 0.90, *ƞ*_p_^2^ = 0.15, *p* < 0.05. According to the Bonferroni calculations, the FRAME condition resulted in larger negativity than the other conditions. According to the Bayesian analysis, the BF_incl._ = 127.177 value indicated extreme evidence of the difference.

In the 226–300 ms epoch, the three-way ANOVA on the TRACK, ISI, and ALL conditions resulted in *F*(2,38) = 4.56, *ɛ* = 0.82, *p* < 0.05, *ƞ*_p_^2^ = 0.19 main effect of condition and condition × side interaction, *F*(2,38) = 6.49, *p* < 0.01, *ƞ*_p_^2^ = 0.25. Positivity in the ERPs in the ALL condition was larger than in the other conditions, and it was smaller in the TRACK condition. The Bayesian analysis indicated anecdotal evidence for the condition difference, BF_incl._ = 5.367 and extreme evidence for lateral difference, BF_incl._ = 9412.63, but no evidence of condition × side interaction, BF_incl._ = 0.194. In the two-way analysis on the FRAME condition, the condition and side main effects were significant, *F*(1,19) = 45.58, *p* < 0001, *ƞ*_p_^2^ = 0.71 and *F*(1,19) = 12.52, *p* < 0.01, *ƞ*_p_^2^ = 0.40, respectively. Deviants elicited smaller positivity, (5.98 vs. 7.79 µV), and ERPs were smaller on the left side (5.72 vs. 8.05 µV). The Bayesian analysis resulted in similar results, extreme evidence for the stimulus and side difference, BF_incl._ = 406.653 and 22,736.057, respectively.

The two-way ANOVA on the difference potentials resulted in significant condition main effect, *F*(3,57) = 7.54, *ɛ* = 0.98, *p* < 0.001, *ƞ*_p_^2^ = 0.28. According to the Bonferroni calculation, the negative difference was larger in the FRAME condition than in the other three ones. The Bayesian analysis resulted in similar result, i.e., extreme evidence for the alternative hypothesis, BF_incl._ = 2.189*10^6^.

## Discussion

The aim of this study was to investigate the effect of various tasks on the ERP signature of detecting task-irrelevant changes (i.e., vMMN). The compared tasks varied in their demand of attention. As a summary of our results, the two sets of analyses (ANOVAs and Bayesian calculations) resulted in robust deviant–standard difference in the FRAME condition (three-stimulus oddball), whereas in the other three conditions, i.e., tracking task (TRACK), target only in the inter-stimulus interval (ISI), and target at any time (ALL) resulted in moderate evidence for vMMN emergence. Although, as Table 1 shows, from the 12 values of the deviant–standard, there was only one positive value, negativities were only in the − 0.14 to − 0.67 μV range. However, studies with similar sample size and design (e.g., Gong et al. 2011; Li et al. 2018; She et al. 2017; Yin et al. 2018) reported vMMN similar to our ISI condition.

Concerning the main question of the present study, i.e., the effect of the ongoing task on vMMN, the results show that central presentation of stimuli together with the temporal separation of task-related and task-unrelated stimuli resulted in a large deviant–standard ERP difference. However, the parafoveal presentation of the task-unrelated stimuli in the other three conditions did not result in detectable vMMN difference. The emergence of a large deviant–standard ERP difference in the FRAME condition corresponds to results of previous studies (e.g., Baus et al. 2021; Kask et al. 2021; Kimura et al. 2011; Kovarski et al. 2017, 2021; Sel et al. 2016; Susac et al. 2010). These studies applied various target stimuli, like having faces with different skin color, spectacle wearers, or marked faces. However, central presentation of the vMMN-related stimuli is not a requirement for vMMN to emerge. Beside the studies at the domain of face processing, parafoveally presented stimuli elicited reliable vMMN (e.g., Berti 2011; Clifford et al. 2010; Czigler et al. 2004; Lorenzo-López et al. 2004; Müller et al. 2012).

We investigated two latency ranges, 150–225 and 226–300 ms. As Fig. 1 shows, in all conditions, the peak of negative component (N1/N170) is within the epoch of the earlier component. N1/N170 is an ERP component characteristic at the onset of visual, and especially facial stimuli (for a review, see Schindler and Bublatzky 2020; Tüttenberg and Wiese 2022). Earlier interpretation of the deviant–standard difference in the range of stimulus-specific (exogenous) negativities proposed that such difference is due to the refractoriness of input neurons (Näätänen and Picton 1987; for a detailed explanation, see May and Tiitinen 2010), in contrast to the deviant-related “genuine vMMN” (e.g., Kimura et al. 2009). O’Shea (2015) proposed that instead of refractoriness, it is a stimulus-specific adaptation process. Within the framework of predictive coding theory, stimulus-specific adaptation means a process of building up a model of the characteristic of expected events (Lieder et al. 2013; Stefanics et al. 2014). Therefore, even if adaptation processes played some role in the difference, a parsimonious interpretation of the results is that the system underlying detection of emotional difference did not require that the faces are involved in the ongoing task. As another and also important point is that in the present study, we involved into the averaging process only standard stimuli followed by a deviant. Accordingly, the averaged standards were always preceded by another standard. In the visual modality, initial stimuli of a sequence elicit fairly large exogenous components, but after the second stimuli, there is hardly any further amplitude decrement (see e.g., Johnston et al. 2017, Fig. 1). Finally, and most importantly, as the results of recent studies (Baker et al. 2021; Johnston et al. 2017) show, increased amplitude in the N1/N170 latency range is a direct consequence of a violated prediction. Therefore, we concluded that even in the earlier period, we registered vMMN, although a small one in the TRACK, ISI, and ALL conditions.

Studies in the field of vMMN to facial stimuli use different set of photography. East-Asian studies applied pictures with standardized East-Asian sets. In European and American studies, a large variation of sets were applied. For example, Astikainen and Heitanen (2009) and Stefanics et al. (2014) applied pictures from the Ekman and Friesen (1976) picture set, Kovács-Bálint et al. (2014) from the Trustworthiness Face Database (Oosterhof and Todorov 2008), and Vogel et al. (2015) applied the Max-Planck Institute of Biological Cybernetics set (Troje and Bülthoff 1996). Furthermore, many studies applied schematic faces. Therefore, it is possible that emotional discrimination among the sets were different.

In light of the present results, the question is the automaticity of processes underlying vMMN. In other words: to elicit vMMN, is it necessary or unnecessary to process attentively deviant events within the stimulus sequence? Although “Everyone knows what attention is” (James 1890, p. 403), in fact “No one knows what attention is” (Hommel et al. 2019). The aim of this study was far from defining attention or to select from the various attempts to define this term. In the field of ERP research, a signature of orienting to unexpected stimuli in the three-stimulus oddball task is a positivity (P3a, for a review, see Polich and Criado 2006) corresponding to the later vMMN range (226–300 ms) of the present analysis. As the scalp distribution in the FRAME condition shows, in this range, the ERP was negative. Kovarski et al. (2017, 2021) obtained similar results. Therefore, even if studies on auditory MMN and vMMN sometimes report that the mismatch component is followed by positivity, the present results did not show P3a emergence. As the attentional issue is unsolved at both theoretical and empirical levels, we suggest the following description for characterizing vMMN: this ERP component is a signature of the detection of events violating task-unrelated sequential regularities.

As limitations of the present study, we investigated a specific type of stimuli, emotional faces. It is possible that in case of other types of stimuli (e.g., different stimulus features, object-related differences, or different perceptual categories), there were different effects of the various tasks. Furthermore, it would be useful to involve an ALL-type of task with central presentation of facial stimuli, because in a study using this paradigm (Kecskés-Kovács et al. 2013), fairly large vMMN emerged. Finally, increased sample size may serve to discriminate among the TRACK, ISI, and ANY tasks, but our purpose in the present study was to use a sample size typical in the field.

## Data Availability

The datasets generated and analyzed during the current study are available here: https://gin.g-node.org/gaalzs/Method.
